# Distribution, Antioxidant Capacity, Bioavailability and Biological Properties of Anthocyanin Pigments in Blood Oranges and Other Citrus Species

**DOI:** 10.3390/molecules27248675

**Published:** 2022-12-08

**Authors:** Paolo Rapisarda, Margherita Amenta, Gabriele Ballistreri, Simona Fabroni, Nicolina Timpanaro

**Affiliations:** Council for Agricultural Research and Economics (CREA), Research Center for Olive, Fruit and Citrus Crops, C.so Savoia, 190, 95024 Acireale, Italy

**Keywords:** anthocyanins, citrus, blood orange, bioavailability, antioxidant activity

## Abstract

Anthocyanins are natural pigments that give a red, purple, and blue color to many plant, flower, fruit, and vegetable species. Their presence within the genus *Citrus* was first reported in 1916, and it is well-known that the red color of the flesh and rind of blood (red or pigmented) oranges (*Citrus sinensis* L. Osbeck) is due to the presence of anthocyanins. They are also present in the young shoots, flowers, and peel of lemon (*Citrus limon* (L.) Burm. f.), citron (*Citrus medica* L.), and other citrus species. Since then, the scientific community has expressed increasing interest in studying their profile and distribution, with many published studies focused on the quali-quantitative pattern in the different vegetative tissues belonging to the genus *Citrus*. Moreover, with the discovery of their relevant antioxidant activity, owing to their ability to capture free radicals, much research has been performed in the last two decades on their radical scavenging power, in vitro and in vivo biological properties, and anticarcinogenic capacity, also focusing attention on their bioavailability for humans. The present work is intended as a comprehensive review of the advances in scientific research on anthocyanin pigments naturally occurring within the genus *Citrus*, including their natural distribution, antioxidant capacity, bioavailability, and biological value and properties. The scientific evidence herein reported can be used to further increase the knowledge of this class of compounds and represents a valuable and comprehensive contribution to promoting anthocyanin-rich citrus fruit consumption as a healthy dietary habit.

## 1. Introduction

Anthocyanins are an extensive group of plant pigments belonging to the flavonoid family. They are responsible for the red, purple, and blue colors in some fruits and vegetables. Within the *Citrus* genus, anthocyanins are found mainly in the flesh and sometimes the rind of orange varieties (*Citrus sinensis* L. Osbeck) called blood or pigmented or red oranges [1]. Moreover, the anthocyanins are even expressed in the young shoots and floral tissues of lemon (*Citrus limon* (L.) Burm. f.), citron (*Citrus medica* L.), and other *Citrus* species [2]. The presence of pigmented stigmas containing anthocyanins in young flowers from the ‘Moro’ orange tree has been recently discovered at a commercial orchard located in Sicily (Italy) [2]. Since it is known that floral tissues from sweet oranges do not accumulate anthocyanin pigments, this is the first time that this phenomenon has ever been reported.

The geographical origin of the *Citrus* species was in southeast Asia, and they were later introduced into Europe [3]. Specific regions include the Yunnan province of southwest China, northeastern India in the Himalayan foothills, and Myanmar [4]. Hodgson [1] has suggested that blood oranges originated from Asia, although it has long been thought that these pigmented varieties derived from a genetic mutation that occurred in the Mediterranean area. Molecular analyses conducted by Butelli et al. [5] demonstrated that most of the current blood orange cultivars grown in China are of direct or indirect Sicilian origin; and an old Chinese blood orange variety named Jingxian [6], which is not widespread because of its poor fruit quality and the presence of many seeds in the fruit, is today the only blood orange of Chinese origin. Therefore, even if blood oranges originated in Asia, the long process of *Citrus* domestication and recent propagation techniques adopted, such as grafting on selected rootstocks or selection of superior branches derived from bud mutations, have led to the spread of a specific phenotype; thus, today blood oranges grown in different areas of the planet all have Mediterranean origins.

The most cultivated of the blood orange cultivars in Italy is the ‘Tarocco’ variety, followed by ‘Moro’ and ‘Sanguinello’ [7,8]. There are also varieties of blood oranges that are not very widespread, such as ‘Sanguigno’, ‘Doppio Sanguigno’, etc., from which it is thought that the ‘Tarocco’, ‘Moro’, and ‘Sanguinello’ varieties derive [9].

Since 1973 the CREA-Research Center for Olive, Fruit and Citrus Crops (CREA-OFA) in Acireale (Italy) has been working on a genetic improvement program for existent blood (red) orange cultivars, also targeting the production of new pigmented citrus hybrids with increased size, ease of peeling, and new original sensorial characteristics [10]. New red-fleshed mandarin-like hybrids have been produced by crossing mandarin (*C. deliciosa* Ten.) or some clones of clementine (*C. clementina* ex Tan.), used as female parents, with different cultivars of blood oranges employed as male parents. Phytochemical studies of these new pigmented citrus hybrids have showed that some traits, such as the anthocyanin profile, were different with respect to their male parent, and in some cases an increase in the expression of some characteristics of the progenies was observed [11,12].

Many studies in recent years have shown that anthocyanins accumulated in leaves, fruits, and other plant tissues play an important role in improving the photoprotective functions and act as reactive oxygen species (ROS) scavengers, light screens, and metal chelators [13,14]. Moreover, studies have demonstrated that certain anthocyanins have antiviral, antibacterial, and fungicidal activities [15,16,17]. The contribution of anthocyanins in plant resistance to biotic stresses is poorly understood compared with that in plant resistance to abiotic stresses. Lin et al. [18] have characterized the function of anthocyanins in protecting fruit from green mold, the major postharvest disease of citrus fruit. Compared with other oranges, ‘Tarocco’ orange, one of the most important blood oranges enriched in anthocyanins, showed reduced susceptibility to the necrotrophic fungus Penicillium digitatum (Pd), which causes citrus postharvest green mold. The fungal infection induces anthocyanin production by activating the expression of several genes in the biosynthetic pathway [19]. As health-promoting plant secondary metabolites, anthocyanins are involved in protection against numerous human diseases that are associated with their antioxidant properties [20]. Blood orange juice has demonstrated an important antioxidant activity owing to the presence of anthocyanin and other phenols [21]. Available research data have revealed that the consumption of blood orange juice may produce positive effects in preventing chronic pathological conditions such as cardiovascular diseases and many types of cancers [22].

In this paper, we review the current knowledge about anthocyanin occurrence in *Citrus* species, and their distribution, antioxidant activity, bioavailability, and biological activity.

## 2. Distribution

### 2.1. Qualitative Aspects

**Figure 1:**
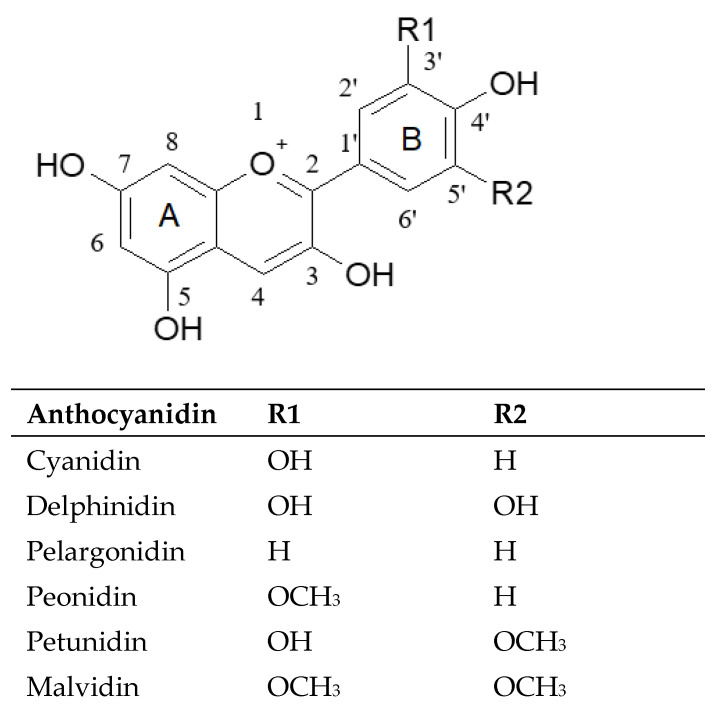
Chemical structure of the most common anthocyanidins.

Anthocyanins are glycosylated derivatives of polyhydroxylated and polymethoxylated flavylium salts, which originate from the flavanones through a reduction reaction catalyzed by determined enzymes [23,24]. The basic structure of anthocyanins is anthocyanidins. The six most common anthocyanidins are: cyanidin, delphinidin, pelargonidin, peonidin, petunidin, and malvidin, with cyanidin being the most widely distributed, followed by delphinidin [25]. Anthocyanin biosynthesis takes place at the endoplasmic reticulum following which they are transported in the vacuoles for cell storage [26,27]. They are usually glycosylated in position 3 but also in position 5 and less frequently in the other positions of ring A and B (Figure 1).

The main sugars are glucose, galactose, and rhamnose and, in many cases, the sugar residue can be acylated with acetic, malonic, or hydroxycinnamic acids.

**Figure 2:**
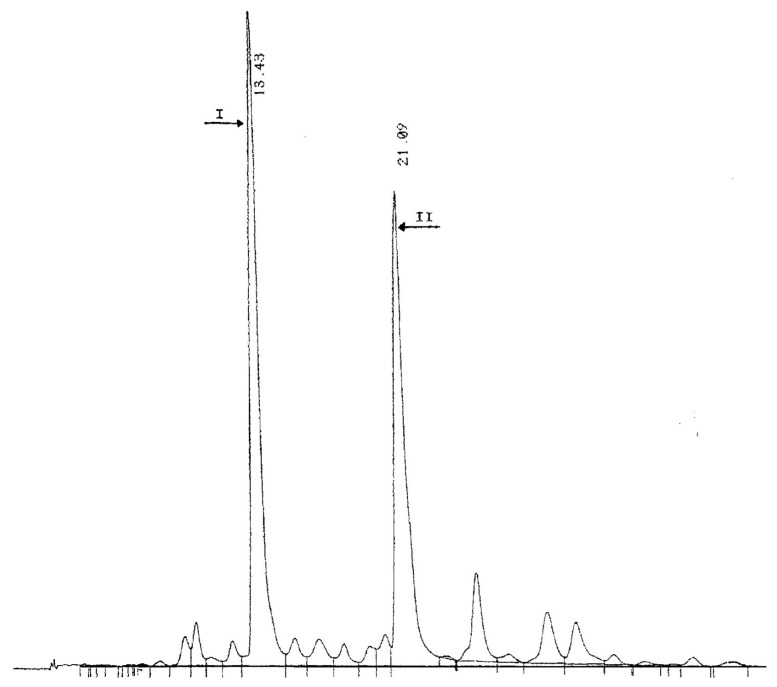
A typical HPLC chromatogram of anthocyanins of blood orange juice (peak I cyanidin-3-glucoide; peak II cyanidin-3-(6″-malonyl)-β-glucoside). Reprinted under CC BY-NC 4.0 from Maccarone et al. [41].

**Figure 3:**
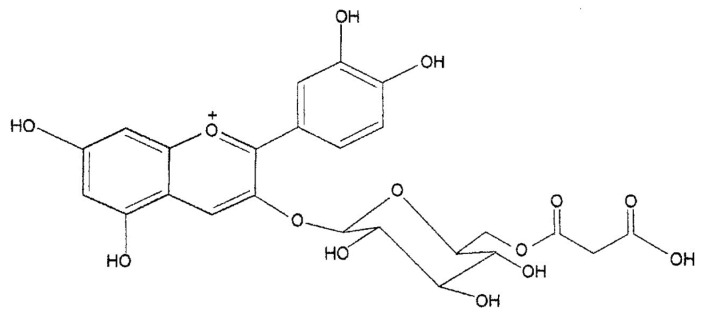
Chemical structure of pigment II: cyanidin-3-(6″-malonyl)-β-glucoside). Reprinted under CC BY-NC 4.0 from Maccarone et al. [41].

The first evidence of the presence of anthocyanins in the genus *Citrus* dates to 1916 when Muriel Wheldale, a British biochemist, in a footnote of her publication “The Anthocyanin Pigments of Plants” stated that anthocyanins are absent in *Citrus* “except in the red-fleshed variety, the so-called Blood Orange” [28]. Subsequently, in 1931, Matlack [29], following his observations on the color variation of the filtered juice after the addition of acids or bases, as well as microscopic observation of the crushed juice sacs in which he identified needle crystals of a deep red to reddish-brown color, confirmed that the pigments present in blood oranges are anthocyanins in nature. The first in-depth studies on the anthocyanins of blood oranges were conducted at the Fruit and Citrus Experimental Station (Italy) in the 1940s. Carrante [30] pointed out that the formation of anthocyanins depends, above all, on the variety, but other factors may influence the production of pigments in orange fruit, such as rootstock, temperature variations between day and night, light intensity, ripening period, and the organic fertilization of the soil. Other studies involved the quantification of anthocyanins in the juices of the different varieties of blood oranges [31] and development of a new method of extracting pigments from the juice [32]. Chandler [33] was the first to identify cyanidin 3-glucoside as the major pigment of the ‘Moro’ variety of the blood orange, with a second pigment, recognized as delphinidin 3-glucoside by chromatographic, chemical, and spectrophotometric methods. In the same paper, the author observed that the main pigments of three other varieties, ‘Tarocco’, ‘Florida’, and ‘Ruby Red’, were chromatographically indistinguishable from those of the ‘Moro’ orange. Kock and Sajak [34], Porretta et al. [35], and Kunkar [36] were the first to point out that cyanidin 3-glucoside is the main component in different blood orange varieties such as the ‘Moro’, ‘Tarocco’, ‘Ruby Red’, ‘Florida’, and Sanguinello varieties, with delphinidin 3-glucoside as a minor component. Finally, Licastro and Bellomo [37], using paper chromatography, separated seven anthocyanins in ‘Moro’ orange juice, five of which were identified as cyanidin 3-glucoside, peonidin 3-glucoside, cyanidin 3,5-diglucoside, petunidin 3-glucoside, and delphinidin 3-glucoside. With the introduction of the high-performance liquid chromatography (HPLC) technique equipped with a UV-Vis detector to separate and identify individual anthocyanins from complex pigment mixtures, a significant step forward in the investigation of anthocyanin distribution in leaves, flowers, and fruits was made. Maccarone et al. [38] were the first to apply this technique to blood orange anthocyanins by separating 14 anthocyanins among the mono and diglucosides in ‘Moro’ orange juice. Cyanidin 3-glucoside was the main component, with lesser amounts of the monoglucosides and diglucosides of delphinidin, petunidin, peonidin, and pelargonidin. Some pigments were acetylated, one of which was identified as cyanidin 3-(4″-acetyl)glucoside. Another study conducted on the most widespread blood orange varieties, including ‘Moro’, ‘Tarocco’ and ‘Sanguinello’, indicated that the distribution of anthocyanins in the different varieties was qualitatively similar [39,40]. The identification of anthocyanins with acetylated sugar in blood orange juice stimulated further research to identify whether they were present in other organic acid moieties. The application of HPLC-mass spectrometry and ^1^NMR spectrometry allowed the elucidation of the structure of a new anthocyanin (pigment II) identified as cyanidin-3-(6″-malonyl)-β-glucoside (Figure 2 and Figure 3) [41].

**Figure 4:**
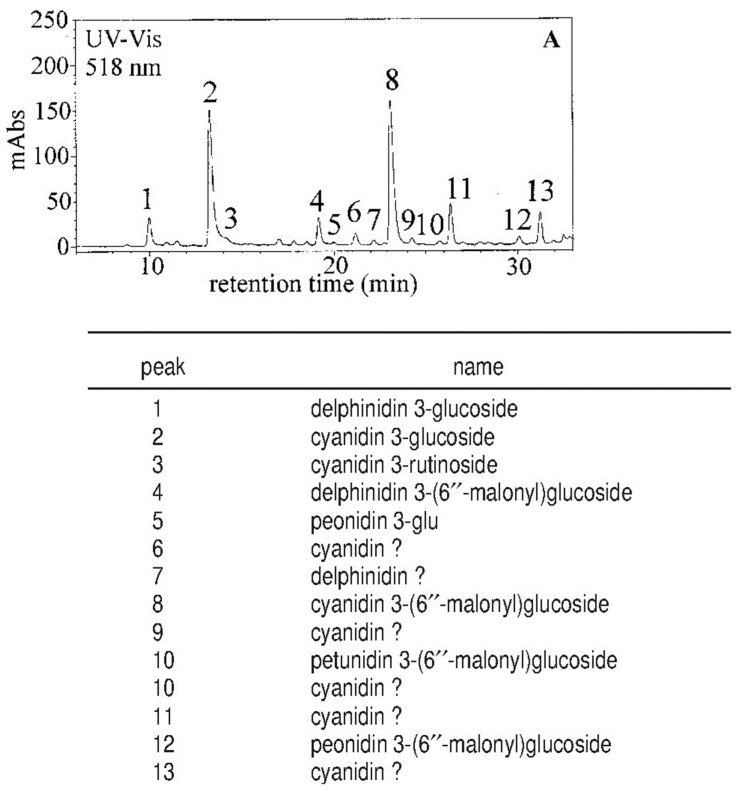
Micro-HPLC-UV-vis chromatogram and identified anthocyanins of ‘Moro’ orange juice. Reprinted from Dugo et al. [42].

Further study performed with micro-HPLC coupled online with an MS detector equipped with an ESI source (micro-HPLC-ESI/MS) [42] allowed detection of 13 anthocyanins in blood orange juice, 5 of them identified for the first time (peonidin 3-glucoside, cyanidin 3-rutinoside, delphinidin 3-(6″-malonyl)glucoside, petunidin 3-(6″-malonyl)glucoside, and peonidin 3-(6″-malonyl)glucoside). Pelargonidin 3-glucoside, previously reported [37], was not detected (Figure 4). Additionally, another five anthocyanins were detected, of which only the aglycon was identified (Figure 4).

Finally, Hillebrand et al. [43] isolated for the first time in blood orange an anthocyanin with an oxalic acid residue identified as cyanidin 3-(6″-dioxalyl)glucoside. With respect to the presence of anthocyanins in other *Citrus* species, Rodrigo et al. [44] pointed out that anthocyanins may also be accumulated in young shoots and fruits and in some floral tissues of lemon (*C*. *limon* (L.) Burm. f.) and citron (*C*. *medica* L.), not being present in the young shoots and flowers of blood oranges. This naturally occurring evidence indicated that anthocyanin biosynthesis in *Citrus* plants may be genotype-dependent and tissue-specific. More recently, the qualitative and quantitative composition of anthocyanin pigments responsible for the red color of fruits and other vegetative and reproductive tissues of *Citrus* plants belonging to different species has been a matter of study and investigation [2]. In this study, the quali-quantitative anthocyanin pattern of blood orange juice and peel was investigated, together with those of the young shoots, flowers, and peel of *C. limon* (L.) Burm. f.; and the young shoots and flowers of *C. limonimedica* Lush, *Citrus medica* L., *Citrus limonia* (Osbeck), and *Citrus meyeri* Y. Tan., finding that the different vegetative tissues share similar qualitative anthocyanin patterns. At the same time, marked quantitative differences were found in their relative percentages, showing tissue- and genotype-specificity. Indeed, while cyanidin 3-glucoside and cyanidin 3-(6″malonyl)glucoside accounted for the largest proportion of the total anthocyanins in the juice and peel samples from blood orange fruits, the dominant pigments in the young shoot and flower samples, regardless of the *Citrus* species involved, were represented by cyanidin 3-glucoside and peonidin 3-(6″malony)glucoside.

Indeed, peonidin 3-(6″malonyl)glucoside represents an O-methylated anthocyanin naturally obtained through methylation and further acylation of a cyanidin-based anthocyanin. This trend suggested that the young shoots and floral vegetative tissues, being simultaneously delegated to guarantee UV photoprotection and attract pollinators, preferentially undergo this kind of reaction. Furthermore, in the same study, the naturally occurring presence of anthocyanins in ‘Moro’ blood orange flower stigmas was detected for the first time with cyanidin 3-(6″malonyl)glucoside, peonidin 3-(6″malonyl)glucoside, and cyanidin 3-glucoside as the major anthocyanins, with the former acylated pigment being the most represented (Table 1).

### 2.2. Quantitative Aspects

The anthocyanin content in the blood orange represents an important index of quality for both the fresh fruit as well as the processing industry. In fact, it is always used as a criterion of the economic evaluation of the product, since the color is a main factor influencing consumer choice. In the past, analytical methods used for other types of red fruit were used to determine anthocyanins in blood orange [45,46,47], but different anthocyanin contents resulting from the application of these methods were noted. Rapisarda et al. [48] performed a quantitative analysis of anthocyanins on a series of blood orange juices according to various spectrophotometric and HPLC methods, and the causes of the different concentrations resulting from the application of these procedures were investigated. Spectrophotometric methods employing aqueous ethanol as a solvent provided an anthocyanin content higher than that determined by HPLC. Discrepancies were ascribed to the use of impure standards and/or unsuitable calibration lines. The most consistent results from the HPLC findings were obtained by a method utilizing water as a solvent and cyanidin 3-glucoside as a standard. The actual concentration of anthocyanins in blood orange juice was remarkably lower than that currently determined by procedures used in the juice-producing factories. The more reliable spectrophotometric procedure appears to be the method described by Rapisarda et al. [49], where aqueous acidic solutions as a solvent and cyanidin 3-glucoside as a standard are used.

The anthocyanin concentration © was calculated by the following equation:

C (mg/L) = (Absp–1 − AbspH4.5) × 484.82 × 1000/24,825 × DF(1)

where the term in parentheses is the difference in absorbance at 510 nm between pH 1.0 and pH 4.5 solutions containing anthocyanins, 484.82 is the molecular mass of cyanidin 3-glucoside chloride, 24,825 is its molar absorptivity (ε) at 510 nm in the pH 1.0 solution, and DF is the dilution factor.

Studies carried out on the anthocyanin content in Italian blood oranges have shown that the different levels of pigmentation are linked to genetic factors. In fact, with the same degree of ripeness, the fruit of the ‘Moro’ cultivar is always more pigmented than the cv. ‘Sanguinello’ and cv. ‘Tarocco’.

Generally, the anthocyanin content trend is ‘Moro’ > ‘Sanguinello’ > ‘Tarocco’. Furthermore, for all three cultivars, the concentration of anthocyanins in the fruits tends to increase during ripening, highlighting a high degree of association between pigmentation level and the soluble solids/acids ratio [7].

**Figure 5:**
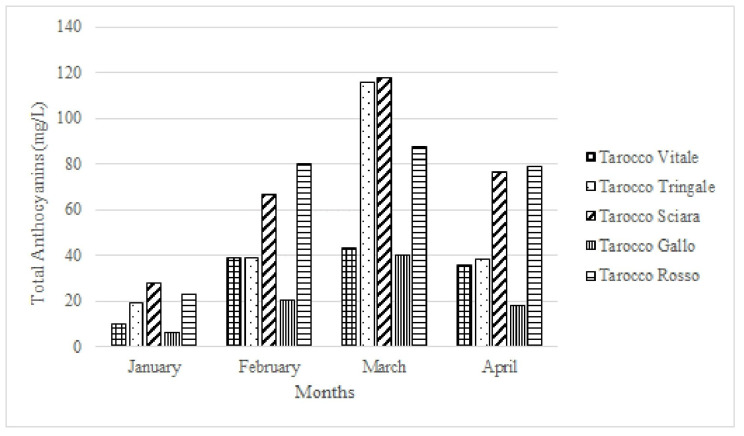
Change in anthocyanin content in different ‘Tarocco’ selections during maturation.

In recent years, ‘Tarocco’ clones with a high anthocyanin level in the fruit, like those of the ‘Moro’ orange, have been selected. Studies conducted on the fruits of five ‘Tarocco’ clones, ‘T. Vitale’, ‘T. Tringale’, ‘T. Sciara’, ‘T. Gallo’, and ‘T. Rosso’, have shown that the levels of anthocyanins increase during ripening with marked differences among the individual genotypes. In February, the fruits of the ‘Tarocco Rosso’ and ‘T. Sciara’ clones were the most pigmented with anthocyanin values between 60 and 80 mg/L, while in March the highest pigment content was found in the ‘T. Tringale’ and ‘T. Sciara’ clones with values of 110 mg/L. The clone with the lowest level of anthocyanins in all the samples was the ‘T. Gallo’, which reached values of 40 mg/L, only in March (Figure 5). The results also indicated that only the ‘T. Tringale’ and ‘T. Sciara’ clones produce juice with an ideal color for processing, given that the standard level of anthocyanins in commercial blood orange juices is equal to 90–100 mg/L [50].

**Figure 6:**
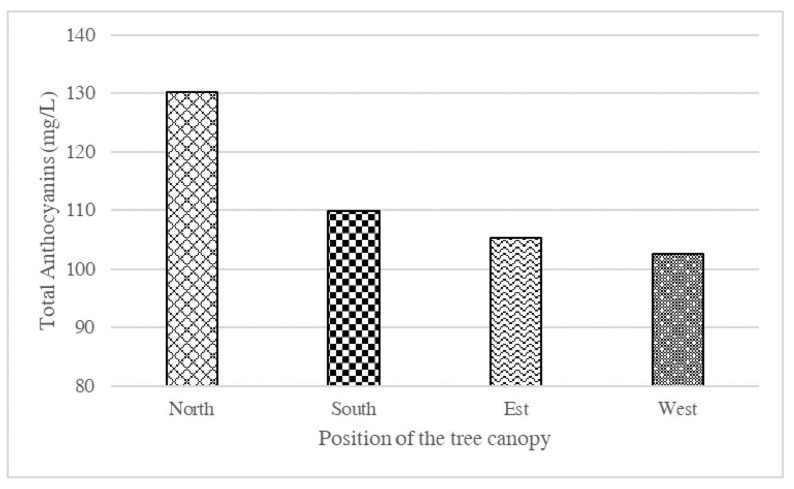
Anthocyanin content in ‘Moro’ fruits collected from different positions of the tree canopy.

In addition to genetic factors, other variables can favor or inhibit the synthesis of anthocyanins in orange fruit. One of the factors that most influence their formation is the light, which favors the biosynthesis of these pigments, even though, in some cases, it accelerates their degradation [51]. Furthermore, the greater or lesser pigmentation of the fruit may be due to its position in the plant. Usually a higher concentration of anthocyanins is found in the fruits exposed to the north (Figure 6), as observed in a study aimed at determining the evolution of pigmentation in ‘Moro’ fruits during the progress of ripening.

Another factor that influences the formation of anthocyanins is the temperature. The development of the red color in the flesh is favored by the different temperatures between day and night. Specific environmental conditions are therefore necessary for the biosynthesis of anthocyanins; in fact, the production of red-fleshed oranges is typical of that area of eastern Sicily located SSW of Mount Etna among the provinces of Catania, Enna, and Siracusa, where these types of thermal variations are most intense. Sicily, therefore, can be considered the homeland for blood oranges as it is the place where selected varieties have found suitable environmental conditions to best express their genotypic characteristics [52].

**Figure 7:**
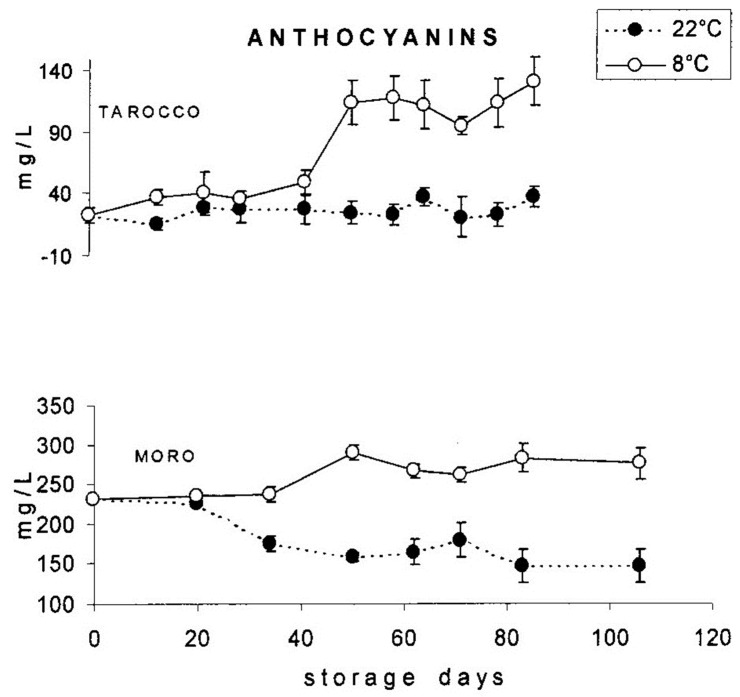
Change in anthocyanin content in the juices of ‘Tarocco’ and ‘Moro’ fruits stored at 8 and 22 °C, respectively. Reprinted from Rapisarda et al. [54].

With respect to the influence of temperature on the development of anthocyanins, studies have been carried out on ‘Tarocco’ and ‘Moro’ fruits stored at 8 and 22 °C (Figure 7), showing that storage at 8 °C determines an increase in the concentration of pigments, especially in the ‘Tarocco’ variety, attributable to the activation of the enzymes involved in the phenylpropanoids metabolism owing to the effect of low temperatures [53,54,55,56,57,58]. Finally, storage at low temperature of blood orange sources of raw material for industrial processes might be a method to increase the concentration of anthocyanins in orange juice [59,60].

## 3. Antioxidant Activity

The great interest in plants and food for their antioxidant properties has increased in recent years. Antioxidants are substances naturally present in food and assumed to be in the diet and generally act on the activities of radical species in a direct way, i.e., providing electrons or hydrogen atoms to turn radicals into non-reactive forms; or indirectly, i.e., by binding metal ions such as copper or iron involved in the catalytic oxidation of lipids [61].

Citrus fruits are powerful sources of antioxidant nutrients such as ascorbic acid, carotenoids, and flavonoids, in addition to other phenolic compounds [62,63]. Various studies have indicated a positive relationship between total antioxidant activity and phenolic compounds or vitamin C content in the fruit and juice of different *Citrus* species and cultivars [64,65,66,67,68,69]. Gardner et al. [70], according with the Miller and Rice-Evans results [71], estimated that the antioxidant activity in orange juices was mainly due (>60%) to vitamin C content.

The antioxidant capacity depends on several factors. The chemical structure of the antioxidants and pre- and post-harvest factors influence the antioxidant capacity of citrus fruits and their derived products [72]. The most relevant compounds in the flavonoid class are anthocyanins, which have a flavylium cation structure. The structure and properties of these phenols, including their antioxidant capacity, are influenced by various factors such as temperature, pH, and solvents that should be controlled to obtain relevant results in terms of their antioxidant activity [73]. Additionally, the glycosylation of anthocyanins has been shown to decrease antioxidant activity and the ability to capture free radicals, compared to the aglycone form, thereby decreasing the power of anthocyanin radicals to delocalize electrons [74]. In general, anthocyanins neutralize reactive radical species by transferring a single electron or by removing the hydrogen atom from phenolic groups. The central component of the antioxidant activity of anthocyanins is represented by the oxidation of phenolic hydroxyl groups—more precisely, the para- and ortho-phenolic groups of B ring (Figure 1), which play a crucial role in the formation of semiquinones and in the stabilization of one-electron oxidation products [75].

Rapisarda et al. [21] evaluated the antioxidant activity of various fresh orange juices obtained from five different *Citrus sinensis* (L.) Osbeck varieties (three pigmented (blood) varieties, ‘Moro’, ‘Sanguinello’, and ‘Tarocco’, and two blond varieties, ‘Valencia late’ and ‘Washington Navel’). Their antioxidant profile (including total phenols, flavanones, anthocyanins, hydroxycinnamic acids, and ascorbic acid) was also determined. The antioxidant activity of these juices was then evaluated by various in vitro tests, and the resulting values were correlated with each one of these classes of antioxidant compounds. The results demonstrated that the antioxidant capability of orange juices may be assigned, in large part, at least, to their total phenols and anthocyanin content. Finally, the blood orange varieties showed higher antioxidant activity than the blond ones.

Another scientific study [76] demonstrated that the new citrus hybrids belonging to the OTA (‘Oroval clementine’ × ‘Tarocco’ orange) and OMO (‘Oroval clementine’ × ‘Moro’ orange) groups containing anthocyanins and/or high levels of flavanones or hydroxycinnamic acids displayed the highest antioxidant activity with respect to hybrids that did not contain anthocyanins. Therefore, one could speculate that the supply of anthocyanins from daily consumption of blood orange juice might provide additional protection against in vivo oxidation of cellular biomolecules. A direct correlation between anthocyanin content and antioxidant activity was also found in a study conducted on ‘Moro’ and ‘Sanguinello’ varieties grown in Turkey [77]. In fact, the antioxidant capacity of ‘Moro’ juice was found to be higher than that of ‘Sanguinello’ juice.

Bonina et al. [78] evaluated the in vitro antioxidant activity of a red orange extract (ROE) obtained from the orange byproducts of three blood varieties (‘Moro’, ‘Tarocco’, ‘Sanguinello’). The results obtained demonstrated the strong antioxidant properties of ROE, with a clear relationship between ROE scavenger efficiency and its content in antioxidant compounds. The antioxidant activity of ROE seems to be related to an overall synergistic effect of the active ingredients of the extract, such as anthocyanins, flavanone glycosides, ascorbic acid, and cinnamic acid derivatives.

The pre-harvest factors include environmental conditions and agronomic management, which act by affecting the level of phytochemicals [79]. Environmental conditions such as soil moisture, radiation, temperature variation, and climatic conditions within a geographical location influence the level of antioxidants in citrus fruits. The difference of temperature between day and night has evident effects on the content of flavonoids, phenolic acid, and anthocyanin, thereby affecting the antioxidant capacity of citrus fruits [80].

Rapisarda et al. [55] reported the responses of five orange genotypes (three pigmented (blood) varieties with different anthocyanin content and two blond varieties) to storage at 6 ± 1 °C for 65 d, focusing on phenolic and vitamin C content and their antioxidant activity. The antioxidant capacity of the juice was assessed by two different in vitro tests (DPPH-scavenging activity, DPPH-SA and inhibition of induced linoleic acid peroxidation, InLAP). The results showed that ‘Moro’ fresh fruit exhibited major DPPH-SA since it contained the highest concentration of anthocyanins, hydroxycinnamic acids, and total phenolics. Thereafter, ‘Ovale’ orange juice showed good initial DPPH-SA because of high flavanones, total phenolics, and vitamin C. There was no significant difference in the initial InLAP values of the different oranges. Finally, antioxidant activity for all the genotypes, measured by two methods, increased significantly during cold storage.

The effect of thermal treatments produced an increase in the main phenolic substances of orange juice, including anthocyanins and total cinnamates, while ascorbic acid underwent a decrease [81]. This phenomenon was probably caused by the thermally induced extraction of antioxidant molecules previously complexed or polymerized and the retention of active principles caused by the inactivation of the enzymes involved in their catabolism. In this case, antioxidant properties were higher in the thermally treated samples.

Post-harvest storage and processing affected the bioactive components and consequently the antioxidant activities of orange juices. Arena et al. [82] evaluated total antioxidant activities (TAAs) of freshly squeezed and processed blood and blond orange juices. Blood orange juices had TAA values higher than blond orange juices, and the TAAs of ‘freshly squeezed’ juices (FSJ) were higher than processed juices. Regarding the processed juices, ‘Reconstituted from concentrate’ (RFC) blood orange juices had higher TAAs than ‘not-from-concentrate’ (NFC) blood orange juices. The difference was due to the increased amount of carotenoid pigments in the serum of RFC juices because of the thermal concentration process. Despite the degradation of anthocyanins during storage, the TAA of NFC and RFC juices remained unchanged up to 60 days at 2 °C, whereas it decreased when RFC juice was stored at 20 °C, in accordance with the observed decrease in ascorbic acid. These results confirmed that antioxidant capacity in orange juice is strictly related to ascorbic acid concentration.

## 4. Bioavailability

For many decades, research and investigations on the beneficial effects exerted by anthocyanin compounds against various diseases, such as antidiabetic, anticancer, anti-inflammatory, antimicrobial, and anti-obesity effects, as well as its effects on the prevention of cardiovascular diseases, have been performed [83]. Eating fruits and vegetables rich in anthocyanins is increasingly recommended by the scientific community as a valuable and healthy dietary habit. As a matter of fact, the bioavailability of the anthocyanin compounds ingested with food has been the target of several studies.

### 4.1. Glucose Transport Receptors as the Main Agents for the In Vivo Absorption of Glycosylated Cyanidins

More than two decades ago, it was believed that only the aglycones of anthocyanins were adsorbed by intestinal cells because of their ability to pass through the gut wall into the bloodstream owing to the lack of a bound sugar residue. Because of the lack of knowledge of a specific enzyme to selectively hydrolyze their glycosidic bonds, it was believed that anthocyanins were scarcely absorbed. The first paper reporting the presence of anthocyanins in their native glycosylated form in plasma appeared in 1999 when Tsuda et al. [84] demonstrated the presence of cyanidin 3-glucoside in the bloodstream of rats, proving that in vivo absorption of glycosidic flavonoids, particularly of cyanidin 3-glucoside, was possible. In 2000, Youdim et al. [85] confirmed these results by measuring plasma levels after oral supplementation of several glycosylated cyanidins, finding that cyanidin-glycosides join the bloodstream by passing through the digestive tract. In this way, Miyazawa et al. [86] and Matsumoto [87] also showed that cyanidin glycosides were detected in their unaltered forms after oral administration in the blood samples of both rats and humans. Later, Netzel et al. [88,89] demonstrated that cyanidin 3-glucoside and cyanidin 3,5diglucoside were excreted in the urine of humans, confirming the previous finding of Cao et al. [90], who first approached the pharmacokinetic absorption of cyanidins, showing that it follows a first-order kinetic mechanism. Further studies by Tsuda et al. [91] also suggested that cyanidin 3-glucoside is partially hydrolyzed by the β-glucosidase reaction in the intestines, as an explanation for the detection of the aglycon in the jejunum.

Successively, Seeram et al. [92] demonstrated in a cell culture study that cyanidin aglycons in plasma are not stable, showing that they are massively degraded to protocatechuic acid (PCA), its main physiological degradation product. This explained the fact that no detection of the aglycon was recorded in plasma, and this was successively confirmed by Vitaglione et al. [93] in an in vivo study on humans. In this way, Ciappelano et al. [94] used an in vitro model to study the absorption of cyanidin 3-glucoside from red orange juice, confirming the crossing of cyanidin 3-glucoside through the intestinal wall in its intact form. Moreover, based on their results, obtained by comparing the use of a standard solution with the use of red (blood) orange juice, they suggested that the rate and kinetic of absorption may be influenced by the food matrix. All of these studies on glycosylated cyanidin absorption suggested that glucose transport receptors were involved in the in vivo absorption of these components. Later, it was demonstrated by Wolffram et al. [95] that the transport of quercetin glycosides by enterocytes can take place thanks to two possible mechanisms, i.e., (i) the transport of quercetin glycosides by the sodium-dependent glucose transporter 1 (SGLT1), and (ii) extracellular hydrolysis by LPH (lactase phloridzin hydrolase), followed by passive diffusion of the aglycone. In 2004, in a comprehensive review paper on the biological activities, absorption, and metabolism of cyanidins, Galvano et al. [96] hypothesized that, based on the similarity of the basic flavonoid structure of cyanidin 3-glucoside to quercetin 3-glucoside, the feasible involvement of the glucose transport receptors on intestinal cell walls in the absorption of cyanidin 3-glucoside was possible.

### 4.2. Anthocyanins as Inhibitors of Glucose Uptake

Felgines et al. [97] conducted an investigation on the absorption of red orange juice anthocyanins in rats, concluding that, presumably, anthocyanin glucosides can interact with the intestinal SGLT1, as previously reported for quercetin. From this evidence came the need to evaluate if, and to what extent, glucose can inhibit intestinal cyanidin 3-glucoside absorption, to verify and validate that SGLT1 is involved in anthocyanin absorption. In this way, Felgines et al. [98] supplemented cyanidin 3-glucoside (pure or from red orange) to rats first by gastric intubation and then using an in situ intestinal perfusion model. They found that the amount of glucose in the perfused solution did not affect the intestinal absorption of pure cyanidin 3-glucoside. These results were also in accordance with a previous in vitro study by Walton et al. [99] in which it was demonstrated that glucose (up to 40 mM) had no significant effect on cyanidin 3-glucoside (5 µM) disappearance from the mucosal solution. As a matter of fact, both studies hypothesized that SGLT1 was not involved in anthocyanin absorption. In contrast, Faria et al. [100] found that anthocyanins interfered with intestinal glucose uptake. Indeed, the authors performed in vitro trials on Caco-2 cells pre-treated for 96 h with an anthocyanin extract from red grape skins (200 µg/mL), detecting the absorption rate of the anthocyanin compounds and gene expression level of SGLT1 and glucose transporter 2 (GLUT2). They found a significant increase in anthocyanin transport and increased expression levels of GLUT2 in pre-treated cells in comparison to controls. Furthermore, they evaluated the putative inhibitory effect of anthocyanins on glucose uptake, finding a 60% decrease in glucose in vitro absorption. Moreover, they tested the effect of ethanol on anthocyanin absorption, finding that it increases anthocyanin in vitro bioavailability, thus suggesting the consumption of ethanol in the same food matrix to improve anthocyanin uptake. These promising results suggested the great potential of anthocyanins as valuable compounds able to inhibit intestinal glucose uptake and increase GLUT2 expression, with relevance for the diabetic population. Subsequently, Alzaid et al. [101] performed a regulation study on human intestinal Caco-2 cells following exposure to an anthocyanin-rich berry extract. Confirming previous findings, they showed that acute exposure (15 min) to berry extract (0.125%, w/v) significantly decreased both sodium-dependent and sodium-independent glucose uptake, also showing SGLT1 and GLUT2 mRNA expression levels significantly reduced after longer-term studies. More specifically, they found that no differential effects on glucose uptake were found for cyanidin aglycone, glucoside, or rutinoside, suggesting that their inhibitory effects might be non-specific and putatively occur owing to steric hindrance rather than for competitive reasons. In any case, this conclusion has been disputed with molecular docking investigations, which suggested that both competitive and steric inhibitions take place [102,103]. More recently, Zou et al. [104] investigated the absorption mechanism of cyanidin 3-glucoside with respect to the role of the SGLT1 and GLUT2 in human intestinal epithelial (Caco-2) cells. In this study the authors showed phlorizin, an inhibitor of SGLT1, and phloretin, an inhibitor of GLUT2, significantly inhibited the absorption of cyanidin 3-glucoside. Furthermore, Caco-2 cells transfected with small interfering RNA to make them specifically deficient in SGLT1 or GLUT2 significantly affected cyanidin 3-glucoside absorption, decreasing its rate. Hence, the authors concluded that SGLT1 and GLUT2 are involved in the intestinal absorption of cyanidin 3-glucoside. Very recently, Baron et al. [105] aimed to better understand the pharmacokinetic properties of anthocyanins from bilberry fruit (Vaccinium Myrtillus) and the relative role of glucose transporters (sGLT1 and GLUT2) in their absorption. Specifically, they measured the absorption of 15 different anthocyanins contained in a standardized bilberry extract (Mirtoselect^®^) with the aim of estimating the bioavailability of each anthocyanin by comparing its relative content in plasma vs. extract. They found that cyanidin 3-glucoside and 3-galactoside, and peonidin 3-galactoside had greater relative abundance in plasma compared with extract, while the most arabinoside-containing anthocyanins were poorly absorbed. Through docking studies, they further demonstrated that sGLT1 has a higher interplay with most of the anthocyanins compared to GLUT2. This behavior was explained by the authors based on the charge of the residues in the binding cavities of the transporters, where GLUT2 preferentially interacts with the neutral tautomer, while sGLT1 can interplay with most of the structural forms. Moreover, they concluded that the binding constant for the GLUT transporters between anthocyanins and glucose should be in contrast because evidence suggested that anthocyanins stop up glucose uptake, but the opposite does not take place. As previously reported, protocatechuic acid (PCA), derived from the B ring of cyanidin 3-glucoside during anthocyanin metabolic processing by enteric bacteria or in chemical reactions, is the main abundant anthocyanin metabolite [84,93]. Based on evidence from the previous studies on anthocyanin bioavailability and absorption through intestinal cells and their involvement in the inhibition of intestinal glucose uptake, a recent study was aimed at investigating the effect of PCA from red orange juice on glucose homeostasis in a murine hepatic cell line and in the liver of C57BL/6 mice [106]. Results of this study showed that PCA improved glucose tolerance and insulin sensitivity in obese mice with early-stage type-2 diabetes, accompanied by high expression levels of glucose transporters glut 1 and glut 4 in the liver. Thus, it was demonstrated that intake of cyanidin 3-glucoside by oral consumption of red orange juice, being responsible for the downstream physiological metabolization and production of PCA, deserves great nutritional interest.

### 4.3. In-Vivo Intervention Studies

Table 2 shows in-vivo intervention studies carried out by supplementation of citrus anthocyanins and their relevant health effects. With the aim of demonstrating a correlation between red orange juice dietary intake and cyanidin 3-glucoside in vivo bioavailability, Riso et al. [107] performed an intervention study on human volunteers who were supplemented with 600 mL/day of red orange juice for 21 days (providing approximately 21 mg of cyanidin-3-glucoside). They showed that cyanidin 3-glucoside levels in plasma significantly increased after the intervention period. At the same time, they observed no effect on antioxidant activity and lipid peroxidation, whereas only lymphocyte DNA resistance to oxidative stress improved. Indeed, even if the study confirmed that cyanidin 3-glucoside can be absorbed as glucoside and its plasma clearance is concluded within 6 h from the intake, it demonstrated that the consumption of blood orange juice for a short period (21 days) does not exert significant influence on markers of lipid oxidation, only producing beneficial effects on lymphocyte resistance to DNA oxidative damage. A further study was performed by Giordano et al. [108] with the aim of evaluating whether a regular and prolonged intake of blood orange juice at nutritional amounts could beneficially affect platelet function and other cellular biomarkers related to cardiovascular risk in healthy subjects. Eighteen healthy subjects (10 men and 8 women) were supplemented for 4 weeks with 1 L/day of either blood (containing 53.09 ± 5.31 mg/L of total anthocyanins) or blond orange juice (that does not contain anthocyanins) as the control. The results showed that urinary excretion of anthocyanins reached increased levels from baseline after 1 week of supplementation, remaining substantially unchanged until the end of treatment. The authors demonstrated that anthocyanins from blood orange juice underwent a rapid and efficient clearance with no plasma accumulation observed, thus the reached levels were insufficient to significantly modify cell markers of platelet and leukocyte activation and interaction. Even though it seemed that the fast in vivo clearance of anthocyanins was not able to produce any long-term effect on cellular biomarker levels, researchers investigated the short-term effect on postprandial low-grade inflammatory response exerted by the simultaneous blood orange juice intake during a fatty meal consumption [109]. In this intervention study a cross-over design was used to supplement 18 apparently healthy subjects with 1 L of either red or blood (containing 53.1 ± 5.3 mg/L of total anthocyanins) or blond (not containing anthocyanins) orange juice, or water (control), during their consumption of a standardized fatty meal that lasted 15 min. Venous blood samples were taken before the meal and after two hours to check for cellular modifications induced by the fatty meal. Results showed that both juices significantly prevented white blood cell increase and release of myeloperoxidase from polymorphonuclear leukocyte, with respect to control, while only the consumption of blood orange juice was able to counteract triglyceride increase in a significant manner in those subjects who were affected by hypertriglyceridemia. Moreover, vascular stiffness significantly decreased only after the meal was consumed in combination with red orange juice. These results showed that the short-term bioavailability of anthocyanins can be responsible for prevention of the low-grade inflammatory modifications induced by a fatty diet. Other studies on blood orange juice supplementation were not able to show its effects on biomarkers of cardiovascular risk in overweight subjects (500 mL/d, providing 50 mg of total anthocyanins, over 28 d) [110] or showed only a moderate reduction in LDL cholesterol concentrations in obese subjects after a 12-week supplementation with 500 mL of blood orange juice per day (containing about 250 mg of total anthocyanins) [111], with the only exception being the study carried out by Buscemi et al. [112], which showed a significant increase in endothelial function and a concomitant decrease in the inflammatory markers after 1 week of supplementation in subjects with high cardiovascular risk (in 2 periods of 7 d each with a 3-d interval, each participant alternatively received 500 mL red orange juice/d, containing 71.3 mg/L of total anthocyanins, and 500 mL placebo/d in a random sequence). Very recently, Li et al. [113] reported a randomized control trial on 15 overweight subjects consuming 200 mL of blood orange juice (containing 2.40 ± 0.13 mg/dL of total anthocyanins) or a sugar-matched control drink (with no anthocyanins) twice daily for two weeks with a washout period of one week. Their results showed a significant 2.01% increase in flow-mediated dilation following consumption of blood orange juice with respect to the control group, highlighting that a two-week consumption of blood orange juice can exert favorable effects on endothelial function in healthy overweight women and men.

Finally, a study demonstrated that ‘Moro’ blood orange juice consumption may inhibit fat accumulation in mice fed a high-fat diet (‘Moro’ juice containing 80.71 mg/L of total anthocyanins and ‘Navelina’ juice, with no anthocyanins, were provided instead of water) [114]. Furthermore, a very recent study showed that a phytoextract rich in anthocyanins recovered from red orange and lemon processing byproducts (red orange and lemon extract was solubilized in water and mice received a daily oral administration containing 120 mg/kg/day of total anthocyanins) can block high-fat diet-induced hyperglycemia and hyperlipidemia in mice [115]. Based on the above, information on the absorption, distribution, metabolism, and excretion of cyanidin 3-glucoside have been largely targeted, evaluated, and validated in the last 20 years, but controversy still exists on the mediated effects of their intake on humans. Much work must still be done to definitively throw light on the in vivo metabolism of cyanidins in humans to find a feasible correlation between cyanidin glycosides-rich food consumption and the occurrence of long- or short-term health benefits.

## 5. Biological Activity

*Citrus* fruits are rich in flavonoids, a wide group of phenolic compounds whose biological activity has been extensively recognized in literature. Numerous research studies have pointed to their antiviral, antimicrobial, anti-inflammatory, anti-ulcerative, and anti-allergenic properties [116,117]. Flavonoids can exert their antioxidant activity in many ways, including radical scavenging such as anti-lipoperoxides and metal chelating agents. Four types of flavonoids (flavanones, flavones, flavanols, and anthocyanins) are present in the genus *Citrus*, the latter being present exclusively in blood oranges and in the young shoots and flower tissues of lemon (*C. limon* (L.) Burm. f.), citron (*C. medica* L.), and other *Citrus* species [2]. These phenolic compounds protect plants exposed to biotic or abiotic stresses such as infections, injuries, UV radiation, pollutants, and other adverse environmental conditions owing to their wide antioxidant properties. These bioactives not only play a significant physiological and ecological role, but they are also commercially relevant because of their wide range of applications in the food, cosmetics, and pharmaceuticals industries. Notably, much *Citrus* flavonoid activity appears to have an impact on blood and micro-vascular endothelial cells, and not surprisingly, the major fields of research on the biological activity of *Citrus* flavonoids are inflammatory and cancerous diseases [117,118].

Anthocyanins are considered to be the most significant subclass of flavonoids because of their high antioxidant activity and other physico-chemical and biological properties [22,117]. This unique group of phytochemicals, consumed in fresh fruits or their derivatives, has been recognized as a highly functional ingredient and for its positive health effects (Table 3).

These bioactive compounds can enhance human health in many ways, and a major one is through their ‘antioxidant’ effects. However, as numerous studies in this field have shown, the health benefits attributed to the compounds present in blood oranges are not only attributable to their antioxidant activity.

New research has demonstrated that these compounds also have anti-inflammatories, anticarcinogens, and many metabolic effects that help protect against diabetes, obesity, risk factors for cardiac disease, and cancerous cell development [22,114,119,121,124,125].

### 5.1. Metabolic Syndrome, Weight Management, and Obesity

The metabolic syndrome is a state characterized by abdominal obesity, high blood sugar and cholesterol, and hypertension, all of which are major risk factors for type 2 diabetes and cardiac disease. The anthocyanin pigments present in blood orange fruits improved insulin resistance, lowered cholesterol and systolic blood pressure, decreasing the risk factors for metabolic syndrome [119]. A 2010 study [114] indicated that blood orange anthocyanins can impair fat cell function and are therefore less likely to be stored as fat. A group of mice were given a regular feeding with the addition of water, blood orange or Washington Navel (blond) orange juice. Another group received a fat-rich diet accompanied by one of the same three beverage alternatives. The group of mice drinking blood orange juice in addition to the standard diet was found to gain less weight with no impact on both blood glucose and lipid levels than those drinking blond orange juice or even water. This was despite the increase in caloric intake from the sugar content of the juice. Moreover, blood orange juice significantly reduced or eliminated weight gain in mice on a fat-rich diet, with a 50% registered decrease in body fat [114]. The intake of anthocyanin-rich blood orange juice also increased the insulin susceptibility in mice through activation of the AMP-activated protein kinase, an established and recognized therapy for diabetes [106]. Treating diabetic patients with an anthocyanin-rich red (blood) orange extract can be therapeutically beneficial to protect them from the complications of diabetes that are caused in part by uncontrolled oxidation of lipids [121]. Pancreatic lipase inhibition, which divides triglycerides into glycerol and fatty acids, is at present a major treatment for obesity. In order to identify other sources for preventing and treating obesity, lipase inhibition using extracts containing anthocyanin was investigated [124]. With regard to inhibition efficacy, the extract enriched with cyanidin 3-glucoside (derived from ‘Moro’ blood oranges) showed the highest in vitro inhibitory efficiency on pancreatic lipase. This result confirmed that anthocyanins are a more effective lipase inhibitor than other natural polyphenols [124].

A follow-up study [125] aimed to establish whether ‘Moro’ orange juice can enhance hepatic lesions in mice affected by dietary-induced obesity. The results demonstrated that ‘Moro’ orange juice neutralizes hepatic steatosis in mice suffering from food-induced obesity, representing a dietary option for the prevention of fatty liver.

### 5.2. Heart Health

The usual consumption of foods high in anthocyanins, including blood (red) oranges, also reduces the risk of heart disease [22,119]. A 2012 study [112] examined the effect of red orange juice consumption on the oxidative stress and inflammatory markers in patients with high cardiovascular risk. The blood flow of the treatment group that received red orange juice was considerably enhanced, and a number of inflammatory biomarkers, including the C-reactive protein, notably declined. These findings suggested an anti-inflammatory effect of red orange anthocyanins that benefits the patient’s cardiovascular system. Dietary intake of anthocyanins may also help protect against hypertension (a relevant risk factor for heart disease) [120]. This study suggested a daily intake of red orange anthocyanins between 12.5 to 15 mg to have a positive impact on reducing and preventing high blood pressure. Furthermore, in healthy patients, the concurrent intake of anthocyanins from red orange juice may prevent the low-grade inflammatory response caused by a fatty meal at cellular and possibly vascular function levels [109], and the supplementation of anthocyanins through blood orange extract is able to reduce oxidative stress, providing protection against its undesirable consequences on human health [122,123].

### 5.3. Anti-Ageing and Photoprotective Effects

Skin is the primary line of defense in the human body, which means it is constantly exposed to a wide range of chemical and physical attacks such as atmospheric pollution and UV rays. A study [126] assessed the protective and anti-ageing effects on the skin of a dosage equal to 100 mg/day of a standardized blood orange extract. This dose amounted to about 3 mg of daily anthocyanins intake. The findings showed a notable decrease in the level of skin rash (redness), with a mean reduction of 40%, as well as pigmentation of the cutaneous spots was observed to decrease from 27 to 7% in subjects exposed to the UV radiation from a solar lamp during the period of supplementation with red orange extract. These experiments showed that blood (red) orange extract was able to compensate for the adverse effects of UV rays as natural sun protection. An analogous study using the same standardized red orange extract (ROE) rich in anthocyanins [82] demonstrated the high antioxidant capacity of ROE in vitro, with a direct relationship between ROE scavenger efficiency and its level of antioxidants. In in vivo experiments, ROE provided effective protection against photo-oxidative skin lesions when topically used immediately after dermal exposure to UVB rays. Moreover, the anti-inflammatory effects of ROE were evaluated on the human keratinocytes that contribute to the physical health of the skin [127]. The results indicated that ROE exhibits anti-inflammatory properties, reducing the adverse consequences of certain skin pathologies such as allergic contact dermatitis, psoriasis, and atopic dermatitis.

### 5.4. Anticancer Activity

Oxidative stress is an event caused by a disproportion between production and accumulation of reactive oxygen species (ROS) in cells and tissues and the ability of a biological system to remove these reactive products [128].

It is responsible for a chronic inflammatory state that plays an important role in neurodegenerative diseases and the development of cancers. Carcinogenesis is a multistep process activated by genetic alterations that modify different signal transduction pathways and cause the gradual transformation of a normal cell into a tumor cell. The signal transduction pathways involved in the formation of tumors often interact with each other, expanding the oncogenic signals necessary for the progression of the malignant form [129]. Available scientific studies have proved the advantageous effects of the presence of anthocyanins in fruits and vegetables in the prevention of tumor diseases (Table 4). Tsoyi et al. [130] investigated the protective effect of anthocyanins on UVB-induced apoptosis. UVB irradiation-induced apoptotic cell death was inhibited by topical application of anthocyanins in hairless mice. This study suggested that anthocyanins may be useful natural products to modulate UVB-induced photoagin

Anthocyanins have been extensively studied for their anticancer characteristics as well as anti-angiogenesis, based on in vitro and cell culture studies and animal models [135]. Endothelial cells are the principal cells involved in the angiogenesis process. Angiogenesis is the key to cancer progress, and it is an important step in the transition of tumors from a benign state to a cancerous one. In studies on the human colon tumor HT-29 cell line, the authors of [131] proposed phosphoglycerate kinase 1 (PGK1) as a possible biomarker of intracellular oxidative damage. Cells exposed to 50 µM H_2_O_2_ for 24 h showed significant expression of PGK1. Additionally, cells treated with delphinidin had attenuated expression of protein. High levels of PGK1 are associated with cancer survival and angiogenesis. These studies proposed that the antioxidant potential of delphinidin could contribute to an anti-cancer approach.

Citrus fruits such as oranges, lemons, tangerines, grapefruits, and limes are widely consumed worldwide. They are rich in bioactive compounds such as carotenoid, folate, vitamin C, limonoids, and flavonoids, which have been demonstrated to have anticancer effects. Various reviews of citrus fruit consumption showed an inverse correlation with the risk of esophageal, gastric, breast, bladder, oral, and pancreatic cancers [132,136,137,138].

Different biological activities of anthocyanins have been studied with the aim of preventing cancer. Grosso et al. [22] discussed the main health-related characteristics of blood (red) oranges that include anticancer, anti-inflammatory, and cardiovascular protection properties, and the effects on health of the main constituents of blood oranges. They specified the mechanism of action of the main components of blood oranges and reported an antimutagenic activity of anthocyanins. Additionally, a cyanidin–DNA copigmentation complex was identified as inhibiting the reverse mutation induced by heterocyclic amines in microsomal activation systems. The antimutagenic action was demonstrated in a study on colorectal carcinogenesis inducted by 1,2-dimethylhydrazine (DMH), confirming a previous study in which juice or extracts of plants or fruits containing high amounts of anthocyanins acted as inhibitors of heterocyclic amine mutagenesis [139]. Forester et al. [133] also described the positive effect of anthocyanin metabolites decreasing cell viability and causing cell cycle arrest and apoptosis in colon tumors. In oral and cervical cancer, the invasion of SCC-4 cells was diminished after the treatment with peonidin 3-glucoside and cyanidin-3-glucoside [66]. Jang et al. [134] studied the effects of anthocyanin on a rat model of benign prostatic hyperplasia (BPH) finding that the injection of testosterone developed prostatic hyperplasia as observed histologically during the tests; it was demonstrated that after anthocyanin treatment the average prostate weight in the BPH-induced group was significantly higher than in the control group, whereas the prostate weights in the anthocyanin-administered groups were significantly lower than in the BPH-induced group. It was concluded that the anthocyanin administration helped prevent this alteration. In addition, apoptotic body counts were significantly higher in groups receiving anthocyanin than in the BPH-induced group. These results suggested that the anthocyanin supplementation may be effective in BPH, and this experiment could be the basis for the clinical application of these compounds. Moreover, some authors have confirmed that the activity of anthocyanins is not mainly due to the compounds themselves; rather, it is the synergetic effect of anthocyanins and other phenolic compounds proving essential for the prevention of diseases. In vitro studies concluded that bacterial metabolism involves the splitting of glycosidic linkages and breakdown of anthocyanidin heterocycle, forming phenolic acids such as protocatechuic, vanillic, syringic, caffeic, and ferulic acids, aldehydes, and their subsequent methyl, glucuronide, and sulfate conjugation [140]. It is conceivable that the observed benefits of consuming anthocyanin-rich foods are due to the complex mixture of metabolites that remain in tissues and biological fluids for a longer time and in higher doses than the parent anthocyanins.

## 6. Conclusions and Future Directions

Even though the presence of anthocyanins in the *Citrus* genus is widespread, the greatest quantities of these pigments are found in blood (red) oranges. These varieties are distinct from other red fruit species because nearly the total amount of anthocyanins is found in the flesh, and only in some varieties (‘Moro’, ‘Sanguinello’ and ‘Doppio Sanguigno’) or some clones of ‘Tarocco’ (‘Lempso’, ‘Rosso’, ‘Vitale’) is it found in the rind. Blood oranges are also characterized by excellent sensory properties (intense and delicate aroma, bright red color, sweet sour taste), higher levels of vitamin C, flavonoids, and hydroxycinnamic acids, compared with blond or common oranges, and by the presence of anthocyanins, also widely recognized as powerful antioxidants.

The three varieties of blood oranges ’Tarocco’, ’Moro’, and ’Sanguinello’ are grown almost exclusively in Italy in an area with a unique microclimate around Mount Etna. Currently, ‘Tarocco’ is the dominant variety, and new ‘Tarocco’ selections with an intense anthocyanin color of the flesh and rind of fruits have been developed.

Changes in lifestyles and eating habits tend to increasingly favor the consumption of orange juice over fresh orange fruit [141]. Currently in Italy, only about 30% of the production of blood oranges is destined to be processed, a very modest amount if we consider that in Brazil and USA about 60% of the production is processed. However, blood orange juice has no problems being placed in markets because it is appreciated by consumers for its peculiar sensorial and health benefits. Therefore, the future of blood oranges is also linked to the development of the juice market and improvement in juice quality. These objectives can be achieved with an increase in the production of blood orange varieties with the highest anthocyanin content and the application of mild technologies for the stabilization of blood orange juice to provide a minimal impact on the fresh orange juice quality, including high hydrostatic pressure (HHP), pulsed electric fields (PEF), supercritical carbon dioxide (SC–CO_2_), etc.

In the past, many papers were published on the in vitro antioxidant activity of anthocyanins, but there are still fewer studies on the in vivo antioxidant activity of anthocyanins, possibly owing to the limited knowledge of their pharmacokinetics. Anthocyanins, through an indirect pathway, enhance endogenous antioxidant defenses, restoring or increasing the activities of antioxidant enzymes such as superoxide dismutase (SOD) and glutathione [142]. It has been demonstrated that the supplementation of a blood (red) orange extract rich in anthocyanin in diabetic patients improves thiol group blood levels on proteins (an indirect measurement of glutathione activity in serum) [122].

The beneficial health effects of blood orange anthocyanins are now well-known, but other blood orange phytochemicals, such as vitamin C, flavanones, and hydroxycinnamic derivatives, work in synergy with anthocyanins to exert therapeutic activities on human diseases associated with oxidative stress. In fact, a blood (red) orange extract (ROE) obtained from the orange by-products of three blood varieties (‘Moro’, ‘Tarocco’, ‘Sanguinello’) used in our abovementioned studies [122], contain 1–3% of anthocyanin, but also 1–2% of hydroxycinnamic acids and 8–15% of flavanones. In the future, it will be important to study the effect of isolated and purified blood orange anthocyanins in comparison with raw blood orange extracts and with the juice as-is, to compare their activities against certain pathologies, including diabetes, obesity, cardiovascular disease, and cancer.

In vivo studies have shown that the bioavailability of anthocyanins in humans is very low, but the literature also suggests that their metabolites may actually be responsible for much of their health-promoting properties. In fact, after the ingestion of blood orange juice, the relevant concentration of PCA in human blood has interesting physiological and nutritional implications. Another important aspect to consider is the measurement of large numbers of metabolites in biological systems after dietary juice or extract supplementation in humans. The widely used methods for such analysis are mass spectrometry (MS) in combination with gas or liquid chromatography (GC-MS, LC-MS) and nuclear magnetic resonance (NMR) spectroscopy, the two methods being largely complementary. These techniques are particularly fast and promising tools for measuring both the administered phytochemicals and their main metabolites produced in the organism in the same experiment.

Finally, the use of modern and efficient extraction technologies, including conventional and advanced techniques such as solvent extraction, microwave-assisted extraction (MAE), ultrasonic-assisted extraction (UAE), subcritical water extraction, and supercritical fluid extraction (SFE), can be useful in producing the relevant quantities of anthocyanins from the industrial by-products of blood orange processing to be used as food colors or plant extracts beneficial for health and disease prevention.

In conclusion, because of their composition and the presence of anthocyanins, blood oranges can be considered a functional food as well as a new source of nutraceuticals.

## Figures and Tables

**Table 1:** Identification and relative amounts of anthocyanins in blood orange juices, peel, and flower stigma. Reprinted from Fabroni et al. [2].

*Citrus sinensis* (L.) Osbeck																
Peak no.	1	2	3	4	5	6	7	8	9	10	11	12	13	14	15	
Compound	cya 3,5-diglu	del 3-glu	cya 3-soph	cya 3-glu	cya 3-rut	pet 3-glu	del 3-(6M)-glu	peo 3-glu	pet 3-(6M)-glu	cya 3-(6M)-glu	cya 3-(6D)-glu	pel deriv.	peo 3-(6M)-glu	cya deriv.	peo deriv.	
Cultivar	Relative composition (%)															Total anthocyanins (mg CGE 100 mL^−1^)
**Juice**																
*‘Tarocco Rosso’*	0.69	2.64	0.29	25.40	2.04	1.22	1.37	0.81	2.18	46.81	6.31	0.76	8.66	0.29	0.53	8.11 ± 0.80 a
*‘Moro nucellare 58-8D-1′*	1.49	4.43	0.65	26.81	3.73	2.06	1.92	1.58	2.50	39.86	5.80	1.80	7.11	0.37	0.61	23.52 ± 1.13 d
*‘Sanguinello Moscato’*	0.14	8.56	1.65	45.01	0.95	0.72	0.68	1.61	1.72	22.35	12.51	0.87	1.95	1.02	0.26	17.64 ± 0.63 c
**Flavedo**																
*‘Moro nucellare 58-8D-1′*	0.56	6.42	0.73	12.95	0.66	1.90	3.82	1.51	8.26	51.49	1.91	4.34	1.60	2.43	1.43	24.00 ± 0.01 d
**Flowers—stigma**																
*‘Moro nucellare 58-8D-1′*	0.11	2.53	0.77	10.30	0.66	4.62	3.01	1.43	3.64	36.73	6.29	8.78	15.30	0.44	5.41	12.23 ± 1.18 b

Mean values (*n* = 3) in the same column followed by different letters are significantly different (*p* < 0.05). Flavedo and flowers-stigma are expressed as mg CGE 100 g^−1^ DW.

**Table 2:** In-vivo intervention studies carried out by supplementation of citrus anthocyanins and their relevant health effects.

Diet Supplementation	Dosage	Duration	Health Effects	References
Red orange juice	600 mL/day	21 days	improved lymphocyte DNA resistance to oxidative stress no effect on antioxidant activity and lipid peroxidation	Riso et al. [107]
	1 L/day	4 weeks	no long-term effect on cellular biomarker levels	Giordano et al. [108]
	1 L	A fatty meal that lasted 15 min	Short-term effects (2 h after meal): white blood cell increase release of myeloperoxidase from polymorphonuclear leukocyte counteraction of triglyceride increase in subjects affected by hypertriglyceridemia decreased kinetic of absorption stiffness	Cerletti et al. [109]
	500 mL/day	28 days	No effects on biomarkers of cardiovascular risk	Hollands et al. [110]
	500 mL/day	12 weeks	moderate reduction in LDL cholesterol concentrations in obese subjects	Azzini et al. [111]
	500 mL/day	2 periods of 7 d each with a 3-d interval	in subjects with high cardiovascular risk: increase in endothelial function decrease in the inflammatory marker	Buscemi et al. [112]
	200 mL twice daily	2 weeks with a washout period of 1 week	2.01% increase in flow-mediated dilation in healthy overweight women and men	Li et al. [113]
	As hydrating medium instead of water	12 weeks	inhibition of fat accumulation in mice fed a high-fat diet	Titta et al. [114]
Red orange extract	120 mg/kg/day of total anthocyanins	8 weeks	reduction of high-fat diet-induced hyperglycemia and hyperlipidemia	Chiechio et al. [115]

**Table 3:** *Citrus* anthocyanins and their positive health effect.

Author(s),. Year	Risk Factor	Effect of *Citrus* Anthocyanins	Disease
Grosso et al., 2013 [22]; Buscemi et al., 2012 [112]; Silveira et al., 2015 [119]; Cassidy et al., 2011 [120].	Blood pressure	Decreased vascular inflammation	Heart disease (atherosclerosis, high systolic blood pressure, high level of chlolesterol, hypertension, ischemic heart)
Grosso et al., 2013 [22]; Silveira et al., 2015 [119].	Cholesterol	Help cholesterol level by raising HDL and lowering LDL cholesterol	Heart disease (high level of LDL cholesterol, stroke)
Grosso et al., 2013 [22]; Bonina et al., 2002, 2005, 2008 [121,122,123].	Oxidation	Decrease oxidation	Heart disease (atherosclerosis, lipid oxidation, oxidative stress)
Grosso et al., 2013 [22]; Cerletti et al., 2015 [109].	Inflammation	Decrease inflammation	Heart disease (atherosclerosis, oxidative stress, vascular stiffness), obesity (high level of abdominal fat)
Talagavadi et al., 2016 [106]; Titta et al., 2010 [114]; Fabroni et al., 2016 [124]; Salamone et al., 2012 [125].	Abdominal fat	Enhanced lipase enzyme activity	Obesity (high blood lipid levels), fatty liver (hepatic steatosis), type 2 diabetes (uncontrolled oxidation of lipids), metabolic syndrome (abdominal obesity, high blood sugar, high cholesterol, hypertension)
Grosso et al., 2013 [22]; Silveira et al., 2015 [119].	Blood levels of glucose	Improved insulin sensitivity	Type 2 diabetes (oxidative damage, high glucose level, high blood pressure)
Bonina et al., 1998 [82]; Puglia et al., 2014 [126]; Cardile et al., 2010 [127].	UV radiations	Photoprotective, anti-ageing	Oxidative damage (skin rash, photo-oxidative skin lesions, allergic contact dermatitis, psoriasis, atopic dermatitis)

**Table 4:** *Citrus* anthocyanins and their anticancer activity.

Author(s), Year	Risk Factor	Effect of *Citrus* Anthocyanins	Disease
Tsoyi et al., 2008 [130]	UVB radiations	Photoprotective,	Photocarcinogenesis, apoptotic cell death
Jang et al., 2008 [131]	Intracellular oxidative damage	Anti-carcinogenic	Colon Carcinoma, angiogenesis
Li et al., 2010 [132]	Inflammation	Reduced risk of prostate or pancreatic cancer	Prostatic or pancreatic cancer
Forester et al., 2014 [133]	Inflammation	Decreasing cell viability, cell cycle arrest and apoptosis	Colon cancer
Jang et al., 2010 [134]	Inflammation	Reduce prostatic hyperplasia	Prostatic cancer
Grosso et al., 2013 [22]	Cell mutation	Anti-carcinogenic, anti mutagenic	Colonic adenocarcinoma, melanoma, vulva carcinoma

## Data Availability

Data available from the authors.
